# Isodorsmanin A Prevents Inflammatory Response in LPS-Stimulated Macrophages by Inhibiting the JNK and NF-κB Signaling Pathways

**DOI:** 10.3390/cimb45020103

**Published:** 2023-02-13

**Authors:** You Chul Chung, Ami Lee, Jin Ah Ryuk, Youn-Hwan Hwang

**Affiliations:** 1 Herbal Medicine Research Division, Korea Institution of Oriental Medicine, Daejeon 34054, Republic of Korea; 2 Korean Convergence Medical Science Major, KIOM Campus, University of Science & Technology (UST), Deajeon 34054, Republic of Korea

**Keywords:** inflammation, NF-κB, chalcone, macrophages, isodorsmanin A, RAW 264.7

## Abstract

Natural and synthetic chalcones exhibit anti-inflammatory, antitumoral, antibacterial, antifungal, antimalarial, and antitubercular activities. Isodorsmanin A (IDA), a chalcone, is a well-known constituent of the dried seeds of *Psoralea corylifolia* L. (PC). Although other constituents of PC have been widely investigated, there are no studies on the biological properties of IDA. In this study, we focused on the anti-inflammatory effects of IDA and evaluated its effects on lipopolysaccharide (LPS)-stimulated macrophages. The results showed that IDA suppressed the production of inflammatory mediators (nitric oxide [NO] and prostaglandin E_2_ [PGE_2_]) and proinflammatory cytokines (tumor necrosis factor-α [TNF-α], interleukin-6 [IL-6], and interleukin-1β [IL-1β]) without cytotoxicity. In addition, it downregulated the mRNA levels of inducible nitric oxide synthase (iNOS) and cyclooxygenase-2 (COX-2) within the treatment concentrations. In our mechanistic studies, IDA inhibited the phosphorylation of the c-Jun N-terminal kinase (JNK), mitogen-activated protein kinase (MAPK), and protected the nuclear factor of the kappa light polypeptide gene enhancer in the B-cells’ inhibitor, alpha (IκB-α), from degradation, thus preventing the activation of the nuclear factor kappa-light-chain-enhancer of activated B cells’ (NF-κB) transcription factor. Our results suggest that IDA is a promising compound for attenuating excessive inflammatory responses.

## 1. Introduction

Inflammation is an essential immune response to a physical or chemical stimulus, such as a wound or bacterial infection, that plays a role in restoring damaged tissues and protecting the body from pathogen invasion [1,2]. However, the inflammatory response causes fever, redness, pain, and swelling, and an excessive response can lead to inflammation-related chronic diseases, such as arthritis, multiple sclerosis, and cancer [3,4]. Inflammation begins when macrophages, which are immune cells, are activated by external stimuli and secrete inflammatory mediators and proinflammatory cytokines, such as nitric oxide (NO) and prostaglandin E_2_ (PGE_2_), as well as interleukin-6 (IL-6), interleukin-1β (IL-1β), and tumor necrosis factor-α (TNF-α), respectively [5,6]. NO is a major inflammatory mediator secreted during inflammation and plays an important role in killing bacteria or removing tumors. However, the overproduction of NO causes tissue and nerve damage, as well as genetic mutations [7]. NO production is related to nitric oxide synthase (NOS). Among the NOS, the neuronal NOS (nNOS) and endothelial NOS (eNOS) are constitutive enzymes that are constantly expressed in neurons and vascular endothelial cells, respectively, whereas the inducible NOS (iNOS) is expressed in macrophages in response to stimuli, such as interferon-γ (IFN-γ), lipopolysaccharide (LPS), and proinflammatory cytokines [8,9,10,11,12]. PGE_2_ is produced by cyclooxygenase-2 (COX-), which inhibits tumor cell apoptosis and induces angiogenesis, leading to tumorigenesis. Macrophages stimulated by LPS or cytokines such as interleukin-1 (IL-1) increase COX-2 expression and promote PG expression, thereby maintaining the inflammatory response [13,14]. The continuous production of inflammatory mediators and proinflammatory cytokines is a major cause of various inflammatory diseases.

The well-known inflammation related signaling pathways such as mitogen-activated protein kinase (MAPK) and nuclear factor kappa-light-chain-enhancer of activated B cells (NF-κB) act as regulators of the expression of proinflammatory cytokines and mediators in LPS-activated macrophages. Normally, NF-κB is bound to the nuclear factor of the kappa light polypeptide gene enhancer in B-cells’ inhibitor (IκB) and is present in an inactive form in the cytoplasm of macrophages. However, upon stimulation with LPS or cytokines, IκB is phosphorylated by IκB kinase (IKK) and degraded by the proteasome through ubiquitination. The isolated NF-κB translocates to the nucleus, where it induces the expression of inflammation-related genes. In addition, the MAPK family, namely, extracellular signal-regulated kinase (ERK), c-Jun N-terminal kinase (JNK), and p38 kinase, can be activated concomitantly or independently through phosphorylation, thereby promoting the expression of inflammatory factors [15,16,17,18,19]. 

Chalcone (1,3-diphenyl-2-propen-1-one) is a simple three-carbon α,β-unsaturated carbonyl system known to exist as a precursor for flavonoid biosynthesis in plants and has been reported to be easy to synthesize in the laboratory [20,21]. Natural and synthetic chalcones have been reported to possess anti-inflammatory, antitumoral, antibacterial, antifungal, antimalarial, and antitubercular activities. Therefore, chalcones have received attention in many studies because of their wide range of biological and pharmacological activities, ease of synthesis, and simple chemical structures [20,22,23].

Isodorsmanin A (IDA; [E]-1-[5-hydroxy-2,2-dimethyl-3,4-dihydrochromen-8-yl]-3-[4-hydroxyphenyl]prop-2-en-1-one) is a chalcone present in the dried seeds of *Psoralea corylifolia* L. (PC) [24]. The biological and pharmacological properties of PC and its constituents have been thoroughly studied [25,26,27,28,29,30,31]. PC is associated with inflammatory signalling pathways such as MAPK and NF-κB; moreover, it also has an influence on the reduction of inflammatory cytokine [32]. In addition, the main constituents of PC are widely studied and are known to have anti-inflammatory properties. For instance, angelicin and psoralen, which are coumarins, are known to cause the inhibition of proinflammatory cytokines expression by regulating NF-κB or MAPK pathways [33,34]. Moreover, bavachin and bavachinin, which are flavonoids, have been reported to have antineuroinflammatory activity, and can inhibit NO and proinflammatory cytokines expression via NF-κB [35,36]. It has also been reported that isobavachalcone and bavachalcone exhibit anti-inflammatory activity in BV-Microglia by reducing NO and proinflammatory cytokines expression [35]. In addition, bakuchiol is known to inhibit NO production via the inactivation of NF-κB transcription [37]. However, to date, there have been no studies on the biological or pharmacological properties of IDA. Therefore, in this study, we aimed to determine the biological activity of IDA, confirm its anti-inflammatory effect, and examine the related molecular mechanisms.

## 2. Materials and Methods

### 2.1. Cell Culture

Murine macrophages (RAW 264.7) were acquired from the American Type Culture Collection (ATCC, Manassas, VA, USA). The cells were maintained and cultured in Dulbecco’s modified Eagle’s medium (DMEM; HycClone, Irvine, CA, USA) containing 100 μg/mL penicillin–streptomycin (P/S; Thermo Fisher Scientific Inc., Waltham, MA, USA) and 10% fetal bovine serum (FBS; Gibco, Grand Island, NY, USA). The cells were incubated at 37 °C with 5% CO_2_ and were sub-cultured every other day.

### 2.2. Measurement of Cell Viability

For measurements of cell viability, Isodorsmanin A, isolated from *Psoralea corylifolia* L., was acquired from ChemFaces (Wuhan, USA, >99 purity). The CCK-8 assay (Cell Counting Kit-8; Dojindo Molecular Technologies, Inc., Rockville, MD, USA) was performed to determine cytotoxicity. Briefly, macrophages (5 × 10^4^ cells/well) were seeded and incubated for 24 h. The cells were pretreated with isodorsmanin A (1.56, 3.13, 6.25, 12.5, and 25 µM) for 3 h and then stimulated with 100 ng/mL of LPS (Sigma–Aldrich, St. Louis, MO, USA) for another 21 h. Thereafter, CCK-8 was added to the microplate well, followed by incubation for 3 h. The absorbance was measured at 450 nm using an enzyme-linked immunosorbent assay (ELISA) microphotometer (BioTek Instruments, Winooski, VT, USA), and the cell viability was analyzed using the measured values. 

### 2.3. Measurement of Inflammatory Mediators and Proinflammatory Cytokines

Macrophages were cultured for 24 h and the IDA (1.56, 3.13, 6.25, 12.5, or 25 µM) samples were pretreated for 3 h before induction with LPS at a concentration of 100 ng/mL. After 21 h, 50 μL of the supernatant was added to 100 μL of Griess reagent (Promega, Madison, WI, USA) mixture (0.1% N-1-napthylethylenediamine dihydrochloride in sterile water, 1% sulfanilamide in 5% phosphoric acid; 1:1) and the absorbance was measured at 450 nm using an ELISA (BioTek Instruments) and was quantified using a standard (sodium nitrate). To determine the levels of PGE_2_, TNF-α, IL-6, and IL-1β, an ELISA from a Quantikine^®^ ELISA Kit (R&D Systems, Minneapolis, MN, USA) was used. The results are presented as the relative percentage of the control (LPS-only group), whereas the percentage of inhibition was calculated using the following formula:

(1) $$\mathrm{Inhibition}\;\mathrm{Percentage}=100-\left(\;\frac{\mathrm{Average}\;\mathrm{control}\;\mathrm{OD}\;-\mathrm{Sample}\;\mathrm{OD}}{\mathrm{Average}\;\mathrm{control}\;\mathrm{OD}}\right)\times 100$$

where Average control optical density (OD) and Sample OD represent the concentration in the LPS-only and IDA-treated groups, respectively.

### 2.4. Nuclear and Cytosolic Extraction

To compare the effect of IDA on NF-κB expression in the cytoplasm and nucleus, RAW 264.7 macrophages were aliquoted into a 60-mm dish, pretreated with IDA for 3 h, and then treated with LPS (100 ng/mL) for different time intervals. Then, the cells were collected, and the nucleus and cytoplasm were separated using the Nuclear and Cytoplasmic Extraction Reagents Kit (Pierce Biotechnology, Rockford, IL, USA).

### 2.5. Western Blotting

Macrophages were cultured and treated with IDA (1.56, 3.13, 6.25, 12.5, or 25 µM) and 100 ng/mL of LPS, after which they were collected and rinsed with Dulbecco’s phosphate-buffered saline (DPBS; Wellgene Inc., Gyeongsan, Korea). The cells were incubated in M-PER^TM^ Mammalian Protein Extract Reagent (Thermo Fisher Scientific Inc.) at 4 °C for 20 min. The cells were lysed using vortex every 10 min, and then the supernatant was separated via centrifugation (400× *g*, 15 min) and collected. The bicinchoninic acid kit (BCA; Thermo Fisher Scientific Inc.) was used to adjust according to the amount of protein contained in each supernatant, and Western blot samples were prepared using equal amounts of protein. Each Western blot sample were loaded and separated on sodium dodecyl sulfate–polyacrylamide gel using electrophoresis. The separated proteins were transferred to a polyvinylidene difluoride membrane. Then, the protein-transferred membrane was then placed in fish serum-blocking buffer (Thermo Fisher Scientific Inc.) at 25 °C for 1 h and incubated at 4 °C overnight with the specific primary antibodies against β-actin (1:5000), iNOS (1:5000), COX-2 (1:5000), p-p38, p38 (1:1000; Thr180/Tyr182), p-JNK, JNK (1:1000; Thr183/Tyr185), p-ERK, ERK (1:1000; Thr202/Tyr204), p-IκB-α, IκB-α (1:1000; Ser 32), and p-NF-κB-p65 (1:1000; Ser 536) (Cell Signaling Technology, Danvers, MA, USA). After incubation, each membrane was washed with TBST (20 mM Tris base, 137 mM NaCl, pH 7.6, and 0.1% Tween-20). Subsequently, the membrane was incubated at 4 °C for 2 h with secondary antibodies (1:1000; Cell Signaling Technology) and then washed three times. The target protein band images were detected using an Enhanced Chemiluminescence Kit (ECL; Bio-Rad).

### 2.6. RT-qPCR Analysis

The total RNA was extracted using the RNeasy Plus Mini Kit (Qiagen, Hilden, Germany). Equal amounts of RNA (2000 ng) were quantified, and cDNA reverse transcription was performed using a cDNA synthesis kit (High-Capacity cDNA Reverse Transcription Kit; Thermo Fisher Scientific Inc.). RT-qPCR was performed using a Taqman Gene Expression Master Mix (Thermo Fisher Scientific Inc.). The TaqMan probes (Thermo Fisher Scientific Inc.) used are listed in Table 1.

### 2.7. Statistical Analysis

The experimental values are presented as the mean ± standard error of triplicate independent experiments. Statistical analysis was performed using one-way analysis of variance (ANOVA) and GraphPad Prism version 9.4.1 (GraphPad Software Inc., La Jolla, CA, USA) for comparisons between the control and multiple groups. The LPS-only and IDA-treated groups at each time interval were compared using two-way ANOVA followed by Turkey’s post hoc test. * *p* < 0.05 and ** *p* < 0.01 indicate significant differences.

## 3. Results

### 3.1. Effect of IDA on Cell Viability

**Figure 1:**
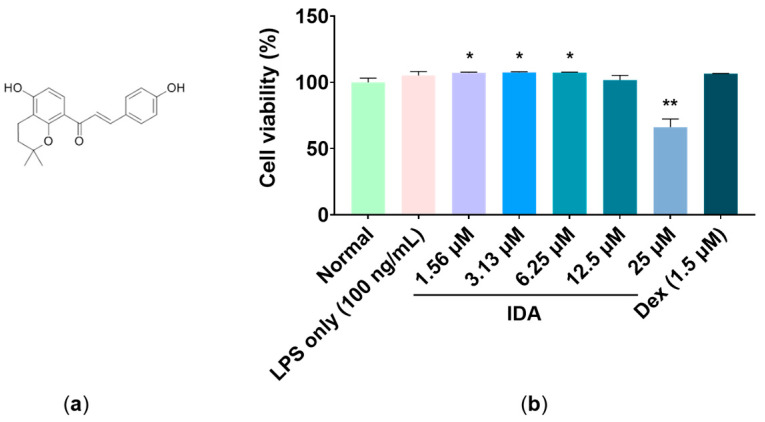
Effect of isodorsmanin A (IDA) on cell viability in RAW 264.7 macrophages: (**a**) The chemical structure of IDA. (**b**) Cells were pretreated with IDA (1.56, 3.13, 6.25, 12.5, or 25 μM) for 3 h and then stimulated with 100 ng/mL lipopolysaccharide (LPS) for another 21 h. The experimental values are presented as the mean ± standard error of triplicate independent experiments. * *p* < 0.05 and ** *p* < 0.01 indicate significant differences vs. the non-treated control (Normal).

To determine the range of cytotoxicity, LPS-stimulated RAW 264.7 macrophages were treated with various concentrations of IDA and a CCK-8 assay was performed to measure their viability. As shown in Figure 1b, there was no significant reduction in viability up to 12.5 μM, whereas viability was significantly reduced by 33.8% when treated with 25 μM compared to that of the untreated control (** *p* < 0.01). Moreover, in comparison to the untreated group, the cells’ viability increased slightly by 7.2%, 7.6%, 7.4%, and 1.8% at IDA treatment concentrations of 1.56, 3.13, 6.25, and 12.5 μM, respectively (* *p* < 0.05). Therefore, once cytotoxicity was established at 25 μM, experiments were conducted at a concentration of 12.5 μM.

### 3.2. Effect of IDA on Inflammatory Mediator Production

**Figure 2:**
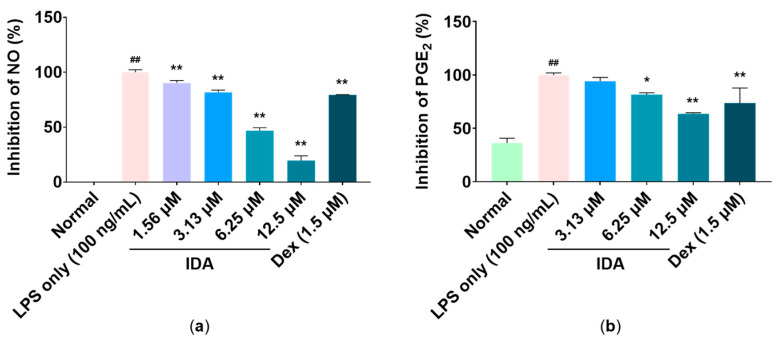
Effect of isodorsmanin A (IDA) on (**a**) nitric oxide (NO) and (**b**) prostaglandin E_2_ (PGE_2_) production in macrophages. Cells were pretreated with IDA (1.56, 3.13, 6.25, or 12.5 μM) for 3 h and then stimulated with 100 ng/mL of LPS for another 21 h. The experimental values are presented as the mean ± standard error of triplicate independent experiments. * *p* < 0.05 and ** *p* < 0.01 indicate significant differences vs. the LPS-only group. ## *p* < 0.01 indicates significant difference vs. Normal group. Dexamethasone (Dex); positive control.

In LPS-stimulated macrophages, the production of NO and PGE_2_ increases rapidly. Therefore, we examined the effect of IDA treatment on LPS-stimulated macrophages. The production levels of NO and PGE_2_ were increased using LPS treatment, whereas the expression levels of inflammatory mediators were significantly decreased using IDA treatment (Figure 2). In particular, 12.5 µM, the highest treatment concentration, markedly reduced NO and PGE_2_ production by 80.4% and 36.5%, respectively (** *p* < 0.01).

### 3.3. Effect of IDA on iNOS and COX-2 mRNA Levels

**Figure 3:**
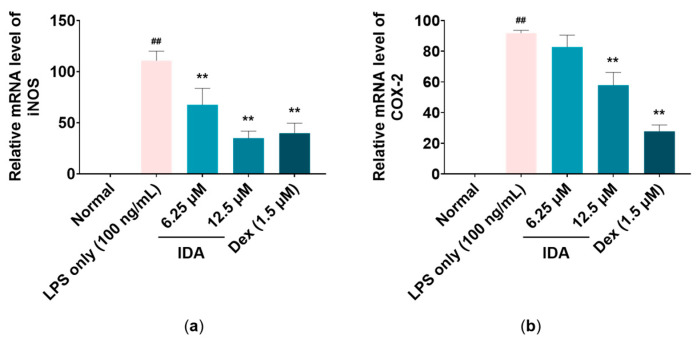
Effect of isodorsmanin A (IDA) on (**a**) inducible nitric oxide synthase (iNOS) and (**b**) cyclooxygenase-2 (COX-2) expression levels in macrophages. Cells were pretreated with IDA (6.25 or 12.5 μM) for 3 h and then stimulated with 100 ng/mL of LPS for another 21 h. The experimental values are presented as the mean ± standard error of triplicate independent experiments. ** *p* < 0.01 indicates a significant difference vs. the LPS-only group. ## *p* < 0.01 indicates significant difference vs. Normal group. Dexamethasone (Dex); positive control.

To confirm the effects of IDA on the mRNA expression levels of iNOS and COX-2, which are related with NO and PGE_2_ production, quantitative reverse transcription polymerase chain reaction (RT-qPCR) analysis was performed. As shown in Figure 3, iNOS gene expression was reduced by 32.5% and 65.1% at IDA concentrations of 6.25 and 12.5 μM, respectively, compared to that in the LPS-only group (** *p* < 0.01). For COX-2, its expression at 12.5 μM was reduced by 42.1% compared to that in the LPS-only group (** *p* < 0.01). The results indicate that IDA downregulates the expression of iNOS and COX-2 mRNA levels.

### 3.4. Effect of IDA on Inflammatory Cytokine Expression

**Figure 4:**
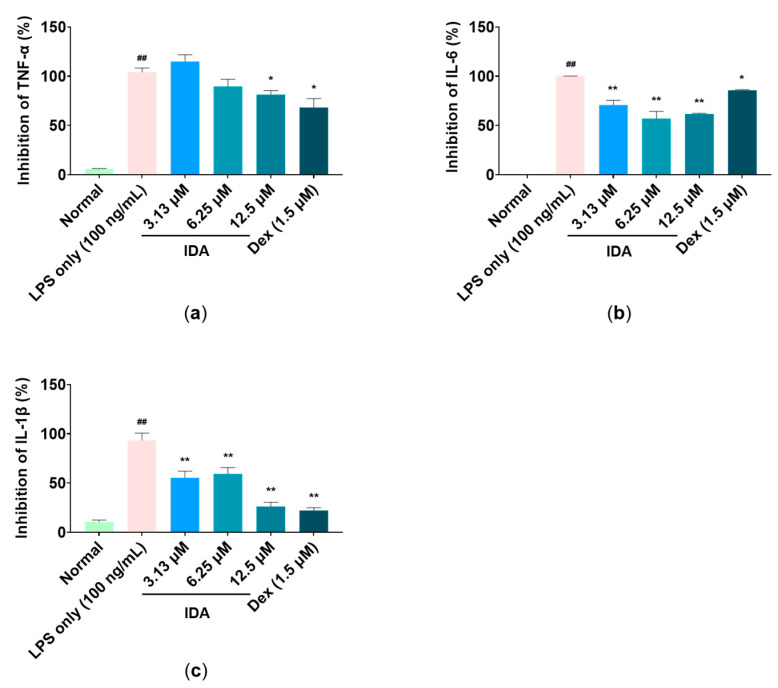
Effect of isodorsmanin A (IDA) on the expression levels of the proinflammatory cytokines: (**a**) tumor necrosis factor-α (TNF-α), (**b**) interleukin-6 (IL-6), and (**c**) interleukin-1β (IL-1β) in macrophages. Cells were pretreated with IDA (3.13, 6.25, or 12.5 μM) for 3 h and then stimulated with 100 ng/mL of LPS for another 21 h. The experimental values are presented as the mean ± standard error of triplicate independent experiments. * *p* < 0.05 and ** *p* < 0.01 indicate significant differences vs. the LPS-only group. ## *p* < 0.01 indicates significant difference vs. Normal group. Dexamethasone (Dex); positive control.

Enzyme-linked immunosorbent assay (ELISA) kits were used to determine the effect of IDA on the expression of proinflammatory cytokines (TNF-α, IL-6, and IL-1β). The results showed that LPS treatment markedly increased cytokine levels compared to the non-treated control. In contrast, each level of proinflammatory cytokines was significantly decreased by IDA treatment in a dose-dependent manner. Notably, 12.5 µM, the highest treatment concentration, inhibited TNF-α, IL-6, and IL-1β expression by 18.6%, 45.1%, and 73.7%, respectively (* *p* < 0.05, ** *p* < 0.01) (Figure 4). 

### 3.5. Effect of IDA on the Phosphorylation of MAPK

**Figure 5:**
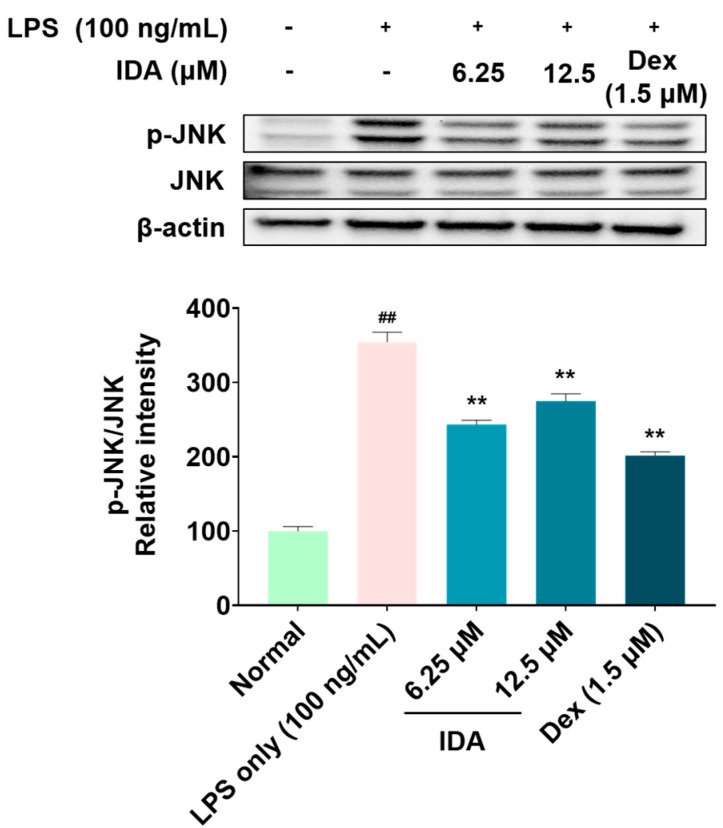
Effect of isodorsmanin A (IDA) on the phosphorylation of c-Jun N-terminal kinase (JNK) in macrophages. Cells were pretreated with IDA (6.25 or 12.5 μM) for 3 h and then stimulated with 100 ng/mL of LPS for another 2 h. Western blot results are from duplicate independent experiments. The results in the graphs are presented as the mean ± standard error from triplicate measurements. ** *p* < 0.01 indicates significant differences vs. the LPS-only group. ## *p* < 0.01 indicates significant difference vs. Normal group. Dexamethasone (Dex); positive control.

Increased phosphorylation of MAPK in macrophages regulates the synthesis of inflammatory factors, thereby increasing the inflammatory response [38]. Therefore, we examined whether IDA affected MAPK phosphorylation in LPS-stimulated macrophages. As shown in Figure 5, IDA treatment inhibited the phosphorylation of only JNK among the MAPK family members; moreover, it did not affect the phosphorylation of ERK and p38 (data not shown).

### 3.6. Effect of IDA on the NF-κB Signaling Pathway

**Figure 6:**
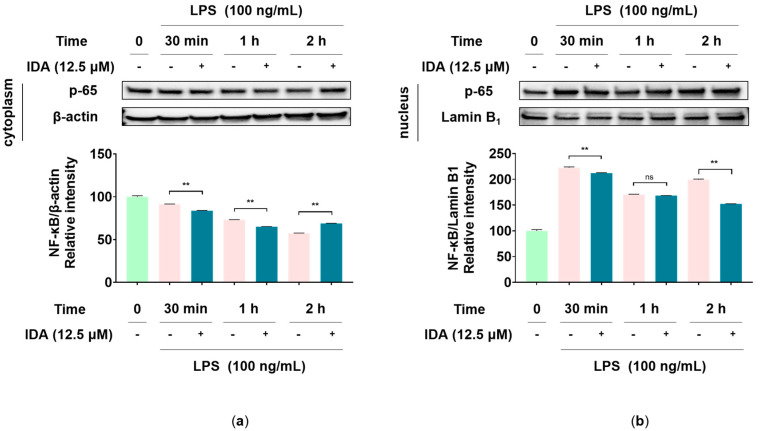
Effect of isodorsmanin A (IDA) on the protein level of the nuclear factor kappa-light-chain-enhancer of activated B cells (NF-κB)-p65 in the (**a**) cytoplasm and (**b**) nucleus of lipopolysaccharide (LPS)-stimulated macrophages. Cells were pretreated with IDA (12.5 μM) for 3 h and then stimulated with 100 ng/mL of LPS for different time intervals (30 min, 1 h, and 2 h). Western blot results are from duplicate independent experiments. The results in the graphs are presented as the mean ± standard error from triplicate measurements. Significance was tested using two-way ANOVA followed by Turkey’s post hoc test. ** *p* < 0.01. ns; not significant.

**Figure 7:**
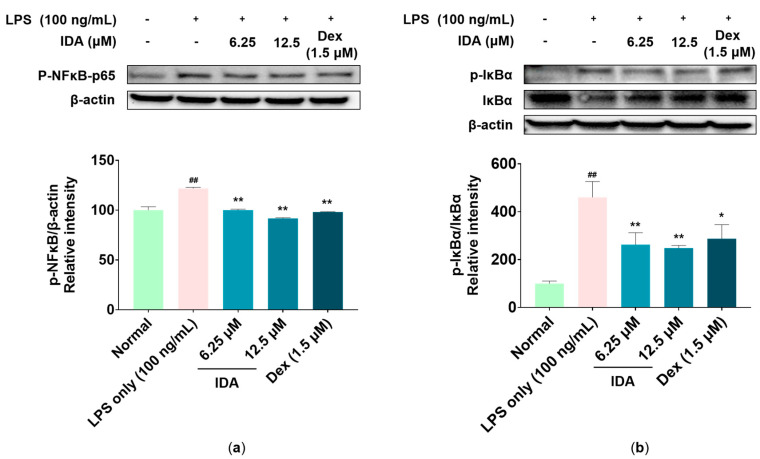
Effect of isodorsmanin A (IDA) on the protein levels of (**a**) phosphorylated (p)-nuclear factor kappa-light-chain-enhancer of activated B cells (NF-κB)-p65, (**b**) nuclear factor of kappa light polypeptide gene enhancer in B-cells’ inhibitor, alpha (IκB-α), and p-IκB-α in macrophages. Cells were pretreated with IDA (6.25 or 12.5 μM) for 3 h and then stimulated with 100 ng/mL of LPS for another 2 h. Western blot results are from duplicate independent experiments. The results in the graphs are presented as the mean ± standard error from triplicate measurements. * *p* < 0.05 and ** *p* < 0.01 indicate significant differences vs. the LPS-only group. ## *p* < 0.01 indicates significant difference vs. Normal group. Dexamethasone (Dex); positive control.

Phosphorylated NF-κB acts as a transcription factor that promotes the synthesis of iNOS, COX-2, and inflammatory cytokines [39]. To determine whether IDA affects NF-κB phosphorylation, we first observed variations in the expression of NF-κB-p65—one of the subunits of NF-κB—over time in the cytoplasm and nucleus. As shown in Figure 6, after 2 h of LPS and IDA treatment, the cytoplasmic expression of p65 was increased compared to that in the LPS-only group, whereas nuclear expression was decreased. As a result of the 2-h LPS treatment in a subsequent experiment, it was determined that NF-κB-p65 phosphorylation, increased by LPS, was inhibited in cells treated with IDA. In addition, it has been reported that LPS-stimulated macrophages induce the phosphorylation and degradation of IκB-α, thereby releasing NF-κB and promoting the activation of the NF-κB pathway [18]. In this regard, our results showed that IDA treatment avoided the LPS-induced degradation of IκB-α by inhibiting its phosphorylation (Figure 7). Therefore, our results suggest that IDA suppresses the expression of NF-κB pathway-related inflammatory factors.

## 4. Discussion

Chalcone, an α,β-unsaturated ketone, is part of the flavonoid family. Chalcones are widely present in plants and exhibit biological activity. Therefore, studies on the efficacy of naturally present and synthetically produced chalcones have recently been conducted. To investigate the anti-inflammatory effects of IDA, a chalcone present in the dried seeds of PC, we examined the expression of inflammatory mediators and proinflammatory cytokines within its non-cytotoxic range. Additionally, RT-qPCR and Western blot analyses were performed to confirm the mechanisms involved in the expression of inflammatory factors. 

Macrophages play an important role in both active and passive immune responses and regulate various inflammatory mediators (including NO and PGE_2_) and proinflammatory cytokines (such as TNF-α, IL-6, and IL-1β) [40,41]. NO plays an important role in killing bacteria and eliminating tumors; however, excessive NO production induces an abnormal inflammatory response and causes inflammatory diseases [7]. In addition, PGE_2_ is generally known as a mediator that induces inflammatory activity through vasodilation, as well as the activation of neutrophils, macrophages, and mast cells in the early stages of inflammation [13]. A recent study showed that treatment with dimethylamino-chalcone inhibited NO production in LPS-induced RAW 264.7 macrophages. In another study, NO and PGE_2_ production was inhibited in these cells through treatment with 2,4,6-trimethoxy-20-trifluoromethylchalcone, a trimethoxy chalcone derivative [42,43,44]. Therefore, we examined the effects of IDA on NO and PGE_2_ production and found that IDA treatment inhibited LPS-induced NO and PGE_2_ production in RAW 264.7 cells without cytotoxicity (Figure 1). iNOS expression is induced by proinflammatory and carcinogenic factors, leading to the production of excess NO. In addition, COX-2 is induced by inflammatory factors, oxidative stress, and cytokines, unlike COX-1, which is constantly expressed in tissues and is known to be involved in PG production [8,9,10,11,12,13,14]. The natural chalcones, Xanthohumol and dihydroxanthohumol, which are isolated from *Humulus lupulus* L., downregulated NO production by suppressing LPS-induced iNOS in murine macrophages [45]. In addition, methoxypenyl- and coumarin-based chalcone derivatives have an anti-inflammatory effect by inhibiting iNOS and COX-2 expression in LPS-induced macrophages [46]. Therefore, we used RT-qPCR to determine whether IDA affected iNOS and COX-2 mRNA expression. The results showed that IDA treatment decreased NO and PGE_2_ production by downregulating iNOS and COX-2 expression (Figure 2). Taken together, these results suggest that IDA reduces the production of NO and PGE_2_ by downregulating iNOS and COX-2 mRNA expression, respectively. 

During an inflammatory response, macrophages induce the expression of proinflammatory cytokines, such as TNF-α, IL-6, and IL-1β, to promote the expression of other inflammatory factors and recruit immune-related cells to the inflamed tissue, eventually promoting the inflammatory response [47]. According to Kim et al., trans-1,3-diphenyl-2,3-epoxypropane-1-one, a chalcone derivative, contributes to the attenuation of the inflammatory response by significantly reducing the secretion of proinflammatory cytokines in LPS-induced RAW 264.7 macrophages [48]. Our results also consistently showed that IDA significantly reduced LPS-induced TNF-α production at 12.5 μM, whereas IL-6 and IL-1β production was significantly inhibited at all treatment concentrations. Therefore, these results suggest that IDA suppresses the inflammatory response by inhibiting proinflammatory cytokine expression.

Signaling pathways involved in various biological responses, including MAPK and NF-κB, play an important role in the inflammatory response, and the regulation of their phosphorylation is closely related to their anti-inflammatory effect [49]. The MAPK pathway, which is composed of three types of kinase (ERK, JNK, p38 kinase), transmits stimuli (e.g., cytokines and reactive oxygen species) from the cell membrane to the nucleus and induces the synthesis of inflammatory factors [50]. Particularly, the phosphorylation of JNK forms the AP-1 transcription factor, which translocates to the nucleus and is involved in the transcription of NF-κB [51]. NF-κB is a transcription factor directly involved in the expression of inflammatory response factors, which is usually present in the cytoplasm in an inactive state, bound to IκB. However, when the IKK complex is activated by LPS stimulation, IKK phosphorylates IκB, freeing NF-κB and allowing it to translocate to the nucleus, where it initiates gene transcription. Therefore, it promotes the inflammatory response by increasing the expression of inflammatory factors [18,52]. Recent studies have reported that several chalcones, namely, 2′-hydroxychalcone, 2′,4-dihydroxy-6′-isopentyloxychalcone, YJI-7, and 2′,4-Dihydroxy-3′,4′,6′-trimethoxychalcone, have inhibitory effects on the inflammatory response by downregulating the MAPK and NF-κB signaling pathways [53,54,55,56]. Our Western blot results indicate that IDA treatment inhibits JNK phosphorylation (Figure 4), but not that of ERK or p38 (data not shown). An examination of the effect of IDA on the NF-κB pathway revealed that p65 expression was upregulated and downregulated in the cytoplasm and the nucleus after LPS treatment, respectively. In addition, p65 phosphorylation was increased; however, this was inhibited in cells treated with IDA. Furthermore, IDA significantly protected IκB-α against degradation by inhibiting its phosphorylation, confirming its effect on IκB-α. These results suggest that IDA downregulates the NF-κB signaling pathway by preventing the phosphorylation and degradation of IκB-α and the resulting NF-κB translocation to the nucleus. Based on these mechanistic studies, IDA suppresses the expression of inflammatory mediators and pro-inflammatory cytokines by downregulating the JNK MAPK/NF-κB signaling pathways. 

In conclusion, this study is the first to investigate the anti-inflammatory activity of IDA, one of the main constituents of PC, in vitro. IDA was shown to have anti-inflammatory effects in macrophages by suppressing inflammatory factor expression via the inhibition of JNK and IκB-α phosphorylation in the MAPK and NF-κB signaling pathways, respectively. These results suggest that IDA has the potential for attenuating inflammation; however, in vivo studies are necessary to evaluate its safety and efficacy in detail.

## Figures and Tables

**Table 1:** Gene name, assay ID, and NCBI reference sequence in quantitative reverse transcription polymerase chain reaction (RT-qPCR).

Gene Name	Assay ID	NCBI Reference Sequence
Inducible nitric oxide synthase (iNOS)	Mm00440502_m1	NM 010927.3
Cyclooxygenase-2 (COX-2)	Mm00478374_m1	NM_011198
β-actin	Mm00607939_s1	NM_007393.5

## Data Availability

Not applicable.

## Sample Availability

The compound isodorsmanin A is available from the authors.
